# Proteostasis of α-Synuclein and Its Role in the Pathogenesis of Parkinson’s Disease

**DOI:** 10.3389/fncel.2020.00045

**Published:** 2020-03-10

**Authors:** Deqiang Han, Wei Zheng, Xueyao Wang, Zhiguo Chen

**Affiliations:** ^1^Key Laboratory of Neurodegenerative Diseases, Ministry of Education, Cell Therapy Center, National Clinical Research Center for Geriatric Diseases, Beijing Institute of Geriatrics, Xuanwu Hospital Capital Medical University, Beijing, China; ^2^Center of Neural Injury and Repair, Beijing Institute for Brain Disorders, Beijing, China; ^3^Center of Parkinson’s Disease, Beijing Institute for Brain Disorders, Beijing, China

**Keywords:** α-synuclein, phase transition, Parkinson’s disease, proteostasis, genetic mutations, inflammation

## Abstract

Aggregation of α-Synuclein, possibly caused by disturbance of proteostasis, has been identified as a common pathological feature of Parkinson’s disease (PD). However, the initiating events of aggregation have not been fully illustrated, and this knowledge may be critical to understanding the disease mechanisms of PD. Proteostasis is essential in maintaining normal cellular metabolic functions, which regulate the synthesis, folding, trafficking, and degradation of proteins. The toxicity of the aggregating proteins is dramatically influenced by its physical and physiological status. Genetic mutations may also affect the metastable phase transition of proteins. In addition, neuroinflammation, as well as lipid metabolism and its interaction with α-Synuclein, are likely to contribute to the pathogenesis of PD. In this review article, we will highlight recent progress regarding α-Synuclein proteostasis in the context of PD. We will also discuss how the phase transition status of α-Synuclein could correlate with different functional consequences in PD.

## Introduction

**Figure 1:**
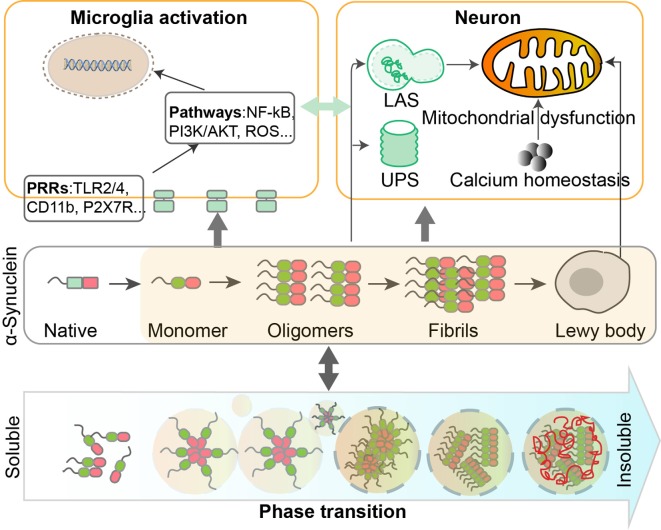
Dynamic regulation and phase transition of α-Synuclein are involved in the pathogenesis of Parkinson’s disease (PD). α-Synuclein consists of three domains and can undergo post-translational modifications (PTMs) such as phosphorylation, SUMOylation, nitration, and O-GlcNAcylation. The transition of α-Synuclein from a monomeric to oligomeric state and further to fibrils is related to the pathological gain of toxicity in PD. Intracellular homeostasis of α-Synuclein is maintained under the surveillance of the ubiquitin-proteasome system (UPS) and lysosomal autophagy system (LAS). Accumulation of α-Synuclein will take place when these degradation systems are damaged. α-Synuclein aggregates could impair mitochondrial functions in human dopaminergic neurons, by altering calcium homeostasis. Different forms of α-Synuclein could activate microglia through different receptors and downstream pathways, leading to inflammatory responses that contribute to neurodegeneration.

Parkinson’s disease (PD) is a common progressive neurodegenerative disorder that affects 1–3% of the aging population (Pringsheim et al., 2014; Kalia and Lang, 2015). The clinical symptoms of PD include resting tremor, rigidity, bradykinesia and postural instability (Jankovic, 2008). One of the neuropathological hallmarks of PD is reduced dopaminergic neurons in the substantia nigra (SN) pars compacta, which causes striatal dopamine deficiency (Obeso et al., 2010; Poewe et al., 2017; Hyung Ho Yoon and Jeon, 2018). Another pathological feature is intraneuronal inclusions, such as Lewy bodies and Lewy neurites in residual dopaminergic neurons. The major constituent of Lewy body is aggregated α-Synuclein (Spillantini et al., 1997; Wakabayashi et al., 2013). Abnormally folded proteins may present with different forms, such as small oligomers, aggregates, and complex inclusions; accumulation of misfolded proteins contributes to the progression of neurodegenerative diseases including PD (Hetz and Mollereau, 2014). Proteostasis is essential in maintaining normal cellular metabolic functions that regulate the synthesis, folding, trafficking, and degradation of proteins. The toxicity of the aggregating proteins is dramatically influenced by the physical and physiological status. In addition, genetic mutations may affect the metastable phase transition of proteins. In addition to playing a central role in the neurodegenerative process of PD, α-Synuclein contributes to the initiation and persistence of inflammatory responses—another important feature of PD. Also detected in Lewy body aggregates in post-mortem brain tissues are lipid vesicles and membrane fragments (Shahmoradian et al., 2019), suggesting that lipid metabolism may be implicated in PD. In this review, we will highlight recent progress in the research area regarding α-Synuclein proteostasis and its role in the pathogenesis of PD. We will also discuss how phase transition and posttranslational modification (PTM) of α-Synuclein may correlate with different functional consequences. Additionally, we will take into account neuroinflammation and lipid metabolism and discuss its interaction with α-Synuclein in the context of PD (Figure 1).

## α-Synuclein Plays a Central Role in the Neurodegenerative Process of Parkinson’s Disease

### Homeostasis of α-Synuclein in PD

α-Synuclein is the major component of intraneuronal protein aggregates in patients with PD and plays a key function in the progression of this disease (Poewe et al., 2017). Intracellular homeostasis of α-Synuclein is maintained under intrinsic surveillance mechanisms including the ubiquitin-proteasome system (UPS) and lysosomal autophagy system (LAS). LAS seems to be more important than UPS in clearing the oligomeric assemblies. A recent study showed that inclusion formation is dependent on the concentration of α-Synuclein, whereas clearance of inclusion is mediated by the autophagy pathway, as revealed by quantitative measurement of the formation and clearance of α-Synuclein inclusions in a yeast model of PD (Perrino et al., 2019). Accumulation of α-Synuclein is largely associated with the impairment of these degradation systems (Xilouri et al., 2013). In turn, abnormal proteins can directly or indirectly interfere with the UPS mechanisms and further affect the function of related pathways, leading to irreversible changes in neuronal protein homeostasis and degeneration (Cookson, 2009; Sato et al., 2018).

### Structure Feature of α-Synuclein Underlies the Pathogenesis of PD

α-Synuclein is normally expressed at high levels in neurons and possibly in oligodendrocytes in the CNS (Asi et al., 2014; Mehra et al., 2019). It is an intrinsically disordered protein that exists in both a soluble and a membrane-bound form in neurons (Mehra et al., 2019). The structure of α-Synuclein includes three domains. N-terminal region (1–60 Aa) that enables the protein to bind to membranes, contains seven conserved repeat sequences which form an amphipathic α-helix (Davidson et al., 1998). Non-amyloid component (NAC; 61–95) containing a highly hydrophobic motif that regulates the oligomerization and fibrillogenesis process is necessary for aggregation of α-Synuclein. The C-terminal tail (96–140) is involved in nuclear localization as well as interactions with other proteins, small molecules, and metals (Eliezer et al., 2001; Ulmer et al., 2005). The pathological gain of neurotoxicity related to α-Synuclein involves multiple biological processes. Initially, soluble α-Synuclein monomers form oligomers, then gradually accumulate into insoluble mature fibrils; eventually, α-Synuclein aggregates into large insoluble fibrils which is more toxic to neurons and can cause cell death and progressive motor impairment (Melki, 2015; Peelaerts et al., 2015; Mor et al., 2016; Karpowicz et al., 2019; Ma et al., 2019). The fibrillar form of α-Synuclein assemblies is capable of promoting aggregation of monomeric α-Synuclein *in vitro* and this phenomenon can spread across cells in a prion-like fashion in cell cultures and animal models (Karpowicz et al., 2019; Ma et al., 2019).

### Phase Transition of α-Synuclein and Its Potential Role in the Pathogenesis of PD

Phase separation occurs when single-phase complexes divide into a concentrated phase and a diluted phase. Eukaryotic cells use phase transition strategies to facilitate the formation of membraneless intracellular territories (Verdile et al., 2019). Through weak intermolecular interactions, multivalent proteins reach a solubility limit to form liquid condensates (Banani et al., 2017; Shin and Brangwynne, 2017). Several proteins undergoing phase transition include intrinsically disordered regions (IDRs). The IDRs often contain prion-like domains (PLDs) and low complexity domains (LCDs; Hughes et al., 2018; Maharana et al., 2018; Wang et al., 2018). Further, the N-terminal domain of α-Synuclein that mediates the aggregation process has two LCDs, indicating that α-Synuclein may, under appropriate conditions, undergo phase separation (Guerrero-Ferreira et al., 2018; Li et al., 2018).

α-Synuclein in monomeric ensembles can exist as distinct conformational phases (Jónsson et al., 2012). Aggregation of abnormally folded proteins is generally considered as a sequential oligomerization process, adding monomers to the already formed nuclei (Serio et al., 2000). The process of aggregation displays an initial lag phase in which precursor clusters assemble spontaneously. To examine such early steps in aggregation, Narayanan et al. (2019) developed a quantitative methodology that employs super-resolution imaging of fixed cells and light-sheet imaging of living cells. They found that mammalian cells have precursor clusters even under normal growth conditions, suggesting that early aggregates behave like condensates.

To further shed light on the early events of aggregation formation of α-Synuclein, recent studies showed that liquid-liquid phase separation of α-Synuclein precedes aggregation by using *in vitro* reconstitution and cellular models. Liquid-like droplets of α-Synuclein generated *in vitro* eventually transition from a liquid to a solid form that contains oligomers and fibrils. Consistently, some aggravation-related factors like low pH, phosphomimetics, and familial PD mutations also promote α-Synuclein liquid-liquid phase separation and its subsequent maturation. Furthermore, *in vivo* evidence demonstrated that liquid droplets of α-Synuclein transform into perinuclear aggresomes under oxidative stress. The phase transition of natural unstructured α-Synuclein and its transformation to an aggregated disease-associated state is closely correlated with the pathogenesis of PD (Ray et al., 2019).

## Toxicity of the Aggregating Proteins Is Dramatically Influenced by Its Physical and Physiological Status

α-Synuclein also undergoes substantial PTMs (Zhang et al., 2019), which include phosphorylation, SUMOylation, Nitration, and O-GlcNAcylation, et al. Toxicity and aggregation of α-Synuclein are largely related to these PTMs.

α-Synuclein can be phosphorylated at serine 129; phosphorylation at this position is toxic and can enhance the formation of α-Synuclein oligomers and accelerate neuronal loss. α-Synuclein can also be modified by PIAS2 that adds a small ubiquitin-like modifier (SUMO) at lysine residues. However, how SUMOylation may influence the property of α-Synuclein remains inconclusive and seemingly contradictory results have been reported. Rott et al. (2017) showed that SUMOylation can promote α-Synuclein aggregation, and meantime inhibit α-Synuclein ubiquitination and reduce its degradation. In contrast, Krumova et al. (2011) reported that SUMOylation can inhibit α-Synuclein aggregation and promote protein solubility. Further studies are needed to address this issue.

Oxidative stress may be another contributor to the pathogenesis of PD. Widespread accumulation of nitrated α-Synuclein in inclusions have been detected by using antibodies specific to nitrated tyrosine residues of α-Synuclein (Giasson et al., 2000). Further studies showed that four locations of tyrosine are susceptible to nitration (Y39, Y125, Y133, and Y136; Giasson et al., 2000; Schapira and Jenner, 2011; Bose and Beal, 2016). Abundant evidence suggests that nitration of α-Synuclein is implicated in the toxicity of aggregates. Nitration of Y-39 speeds up the oligomerization of α-Synuclein. Interestingly, a mutation of this site to cysteine residue leads to high levels of fibrillation (Zhou and Freed, 2004; Danielson et al., 2009). Nitrated α-Synuclein in the form of a monomer or dimer accelerates fibril formation and can seed the fibrillation of unmodified α-Synuclein. Further study showed that nitration-induced α-Synuclein oligomerization involves interactions between the N- and C-terminal regions of different α-Synuclein molecules. Nitration on the N- or C-terminal regions impact the order of oligomerization; for example, only dimers are formed when Y39 is not available for nitration (Burai et al., 2015). Another study showed that nitration of α-Synuclein can stabilize the formation of lower molecular weight oligomers, resulting in decreased fibril formation (Hodara et al., 2004). In all, nitration may be part of the complicated regulatory mechanisms that control the proteostasis of α-Synuclein (Burai et al., 2015).

O-GlcNAcylation is a dynamic process, in which GlcNAc is transferred by O-GlcNAc transferase (OGT) from UDP-GlcNAc to the serine and threonine residues, and subsequently removed by O-GlcNAcase (OGA; Hart et al., 2007). α-Synuclein can be O-GlcNAcylated at nine different positions (Marotta et al., 2015). Proper O-GlcNAcylation of certain proteins prevents their aggregation, and loss of this modification is a contributing factor in the development of neurodegenerative diseases. It has been verified that the O-GlcNAcylation of α-Synuclein stabilizes the monomeric state of proteins and alters the structure of α-Synuclein aggregates (Marotta et al., 2015; Zhang et al., 2019). In general, O-GlcNAcylation inhibits the aggregation of α-Synuclein. Furthermore, α-Synuclein with three O-GlcNAc modifications can prevent the aggregation of unmodified proteins. O-GlcNAcylation of α-Synuclein peptide also inhibits the toxicity of extracellular α-Synuclein fibrils (Levine et al., 2019).

## The Order of Oligomerization and Toxicity

The transition of α-Synuclein from a monomeric to oligomeric state leads to a pathological toxic function in PD. α-Synuclein as monomers interacts and regulates ATP synthase to augment the efficiency of ATP production under physiological conditions (Ludtmann et al., 2016). In contrast, α-Synuclein in the state of beta sheet-rich oligomers interacts with mitochondrial proteins such as ATP synthase and disturbs complex I-dependent functions. Interaction with oligomers induces oxidation of the beta subunit of ATP synthase and peroxidation of mitochondrial lipid. These events further augment the likelihood to form permeability transition pore (PTP) which is toxic to cells (Ludtmann et al., 2018). α-Synuclein fibrils also cause neurotoxicity and cell death by activating nitric oxide synthase (NOS), leading to DNA damage and polymerase-1 (PARP-1) activation. Reciprocally, PAR accelerates the fibrillation of α-Synuclein (Kam et al., 2018). It seems that different states of α-Synuclein exert various, even opposing effects in cells. The soluble monomers are harmless, whereas oligomers and fibrils are toxic, although the extent of toxicity may be different. Exactly how the order of oligomerization and change of the configuration may impact on the toxicity of α-Synuclein requires further studies.

## Proteostasis of α-Synuclein and Inflammation Contribute to Early Pathogenesis of Parkinson’S Disease

Microglia are the major abundant CNS-specific immune cells that participate in the maintenance of brain homeostasis through mediating inflammation and/or phagocytosis. Chronic microgliosis is considered a pathological feature of PD, and the levels of inflammatory factors secreted by microglia correlate with the progression of PD (Labzin et al., 2018). Different forms of α-Synuclein exhibit distinct effects in triggering microglial phagocytosis and inflammation. Rather than oligomers of α-Synuclein which only induce upregulation of IL-1β (Krashia et al., 2019), fibrillar α-Synuclein is able to elicit strong pro-inflammatory responses in a microglial cell line (Gustot et al., 2015; Hoffmann et al., 2016; Zhou et al., 2016). Fibrillar α-Synuclein can activate NLRP3 inflammasome which leads to the cleaving of pro-inflammatory cytokines, such as IL-1β and IL-18 (Zhou et al., 2016; Chatterjee et al., 2020; Haque et al., 2020). Also, using a rat model that overexpresses human α-Synuclein, *Krashia et al*. found that α-Synuclein-induced inflammation precedes nigral degeneration, and administration of resolving D1, a potent lipid mediator that can resolve inflammation to promote restoration of tissue homeostasis, prevents neuronal dysfunction and motor deficits (Krashia et al., 2019). Multiple receptors and pathways are responsible for inducing pro-inflammatory responses in microglia in PD (Table 1). Both TLR2 and TLR4, whose expression levels are increased in PD patients and MPTP-administrated models, can be activated by α-Synuclein to induce sterile inflammation in PD (Kaur et al., 2017; Ferreira and Romero-Ramos, 2018). The oligomer of α-Synuclein can bind to the P2X7 receptor to activate the PI3K/AKT pathway in BV2 cells, a microglial cell line. α-Synuclein aggregates can also be internalized in autophagosomes *via* FcγR on microglia, which leads to activation of NF-kB pathway (Cao et al., 2012). CD36 is possibly involved in α-Synuclein-induced microglial activation but the mechanism remains elusive (Ferreira and Romero-Ramos, 2018). Prostaglandin E2 receptor subtype 2 (EP2) on microglia seems to play a critical part in neurotoxicity caused by α-Synuclein aggregation, based on *in vivo* and *in vitro* evidence (Jin et al., 2007). Aggregated nitrated α-Synuclein can induce ROS production from microglia, which is inhibited by blockade of potassium channels (Thomas et al., 2007). In turn, activated microglia and inflammation might promote α-Synuclein misfolding and aggregation. Wang et al. (2016) reported that inflammation-induced caspase 1 activation directly cleaves wild-type α-Synuclein, and the truncated α-Synuclein is more prone to aggregation and leads to toxicity in a neuronal PD cellular model. The effect of immune cells and inflammation on the spread of prion-like α-Synuclein remains largely unexplored. On the one hand, spread of α-Synuclein is determined by the dynamic net sum of seeding, propagation, and phagocytosis of fibrillar α-Synuclein, and microglia are beneficial in a sense that they can facilitate clearance of α-Synuclein aggregates and damaged neurons. On the other hand, inflammation-induced reactive species might have a negative influence on the conformation changes of α-Synuclein; but the detailed mechanisms require further investigation.

**Table 1:** Receptors on microglia that are involved in α-Synuclein-induced responses.

Receptor	α-Synuclein phase	Pro/anti-inflammation	Description	Reference
CD14 and TLR4	Monomer	Pro-inflammation	Mediating phagocytosis of α-Synuclein	Kitchens (2000), Muroi et al. (2002), Stefanova et al. (2011) and Fellner et al. (2013)
TLR1 and TLR2	Oligomer	Pro-inflammation	Relating to α-Synuclein toxicity and inflammation	Klegeris et al. (2008), Wilms et al. (2009), Prabhakaran et al. (2011), Kim et al. (2013) and Daniele et al. (2015)
CD36	Monomer	Pro-inflammation	Mediating oxidative stress	Ferreira and Romero-Ramos (2018)
P2X7R and eATP	Oligomer	Pro-inflammation	Mediating oxidative stress	Jiang et al. (2015)
CD11b/CD18	Oligomer/fibril	Pro-inflammation	Mediating oxidative stress	Wang et al. (2015)
EP2	Oligomer	Pro-inflammation	Regulating α-Synuclein aggregation and associated neurotoxicity	Jin et al. (2007)
Ion transport channel	Nitrated α-Synuclein	Pro-inflammation	Mediating oxidative stress	Thomas et al. (2007)
FcγR	Fibril and IgG	Anti-inflammation	Preventing hyper-activation and inducing SHP-1 activation	Smith and Clatworthy (2010), Choi et al. (2015) and Chauhan et al. (2017)
MHC II	Peptides	Anti-inflammation	Inducing α-Synuclein degradation	Sulzer et al. (2017)

## The Role of Lipid Metabolism and Its Interaction with α-Synuclein in the Pathogenesis of PD

Accumulating evidence has suggested that lipid metabolism and its interaction with α-Synuclein are implicated in many aspects of PD pathogenesis. Upon binding of α-Synuclein to synthetic lipid membranes in an *in vitro* test, α-Synuclein undergoes a structural transition from random coil to alpha-helical secondary conformation (Davidson et al., 1998). α-Synuclein binds to small vesicles containing acidic phospholipids preferentially instead of to those with a net neutral charge. The membrane-bound form may have a higher aggregation propensity than the cytosolic form, and membrane-bound α-Synuclein can generate nuclei which are able to seed the more abundant cytosolic form (Lee et al., 2002). Interaction with the membrane is in agreement with the proposed biological functions of α-Synuclein, such as regulation of synaptic plasticity. The binding affinity of α-Synuclein to model membranes is much higher when the membrane is in a fluid phase vs. in a gel phase (Galvagnion et al., 2016). The solubility, but not the fluidity, determine the magnitude by which membrane prompts fibril formation of α-Synuclein (Galvagnion et al., 2016). This evidences demonstrated that the chemical properties of lipids determine the balance between functional and deleterious interactions of α-Synuclein with lipid membranes, allowing for a deeper understanding of how this interaction may contribute to neurodegeneration. These data implicate a possible way of involvement of inflammation in aggregate formation since inflammation-induced reactive species can directly alter the property of membranes. Another factor that may influence membrane property is aging. Hallett et al. reported, in the aging brain, an aberrant association between α-Synuclein and dopamine vesicular membrane, which was concurrent with synaptic destabilization (Hallett et al., 2019). However, overexpression of α-Synuclein without lipid deregulation does not result in the otherwise observed abnormality, suggesting that the aberrant association is lipid-dependent (Brekk et al., 2018). Lipid trafficking also seems to be involved in PD pathogenesis. Lipids are transported by Rab proteins. Chung et al. (2009) found that Rab3b is more highly expressed in A10 vs. A9 dopaminergic neurons, which could be one reason accountable for the relatively greater vulnerability of A9 neurons compared with A10 neurons; overexpression of Rab3b in A9 neurons in rats confers a protective effect and leads to improved motor functions in a PD model. In another study, overexpression of a different Rab protein, Rab1a, normalizes expression of α-Synuclein in patient neurons on a genetic background of SNCA triplication, possibly through enhanced trafficking to lysosomes (Mazzulli et al., 2016). The study points out an interesting approach to cope with the accumulation of α-Synuclein in PD.

## Crosstalk Between α-Synuclein and Mitochondrial Dysfunction

Multiple studies have demonstrated that α-Synuclein aggregation and mitochondrial dysfunction are both important in the pathogenesis of PD. Accumulating data showed that α-Synuclein aggregation and mitochondrial defects may have a bidirectional interaction. α-Synuclein interacts with mitochondria by specifically binding to the TOM20 receptor, inhibiting mitochondrial protein import machinery, and impairing mitochondrial functions (Di Maio et al., 2016). α-Synuclein aggregation in mitochondria impairs complex I in human dopaminergic neuronal cells, consequently interfering with mitochondrial functions (Devi et al., 2008). Additionally, it was suggested that soluble, prefibrillar α-Synuclein, but not α-Synuclein monomers, impairs the retention of Ca^2+^ in mitochondria, and induces mitochondrial depolarization and swelling, leading to Ca^2+^ dependent mitochondrial dysfunction (Luth et al., 2014). On the other hand, mitochondrial defects result in oligomerization and accumulation of α-Synuclein, which in turn aggravates the dysfunction of mitochondria. Growing evidence suggests the involvement of α-Synuclein in the dynamics of mitochondria, such as mitochondrial fission, fusion, and mitophagy. Overexpression of α-Synuclein in *Caenorhabditis elegans* and cultured cells reduces mitochondrial fusion, resulting in fragmentation of mitochondria (Kamp et al., 2010). And these fusion deficits and mitochondrial fragmentation are rescued by overexpression of PINK1, PARKIN or DJ-1 (Kamp et al., 2010). However, the detailed picture illustrating the interaction between α-Synuclein accumulation and mitochondrial dysfunction remains obscure and requires further investigation.

## Mechanisms Underlying the Connection Between α-Synuclein Accumulation and Genetic Factor-Associated PD

The majority of PD are sporadic, and around 5–10% of PD are familial, presenting with monogenic forms of the disease (Rocha et al., 2018). More than 20 PD-related genes have been identified, including SNCA, leucine-rich repeat kinase 2 (LRRK2), glucocerebrosidase (GBA), PINK1, DJ-1 and Parkin (Li et al., 2014; Kalinderi et al., 2016; Balestrino and Schapira, 2018).

Studies showed that accumulation of insoluble α-Synuclein plays a significant role not only in the neurodegenerative process of sporadic PD but also in familial PD; the interaction between α-Synuclein and mutant genes contributes to neuronal death, dysfunction, and loss of connectivity (Stojkovska et al., 2018). The genetic mutations are mostly involved in α-Synuclein aggregation or clearance pathways, often leading to early-onset PD.

Mutations in the GBA is the single largest risk factor associated with PD (Balestrino and Schapira, 2018). A lot of studies have indicated a key role for α-Synuclein in the pathogenesis of GBA-PD. GBA encodes lysosomal enzyme GCase that catalyzes the hydrolysis of glucosylceramide (GlcCer), and mutation of this gene normally results in dysfunction of the autophagy-lysosomal pathway (Robak et al., 2017). Since degradation of α-Synuclein partially depends on autophagy, lysosomal dysfunction caused by GBA mutation could lead to α-Synuclein accumulation and aggregation (Vogiatzi et al., 2008). Induced pluripotent stem cell (iPSC)-derived neurons from GBA-deficient PD patients show a reduced GCase activity and higher levels of GlcCer as well as α-Synuclein aggregation due to lysosomal defects (Schöndorf et al., 2014). On the other hand, α-Synuclein aggregation, in turn, inhibits GCase activity, and the toxic oligomeric form of α-Synuclein is stabilized by an increased level of GlcCer, the substrate of GCase (Mazzulli et al., 2011). These data demonstrated that α-Synuclein and GCase may form a bidirectional pathogenic loop, participating in a self-propagating feedback process that eventually leads to neurodegeneration (Mazzulli et al., 2011). In addition, GBA mutations can elevate the level of α-Synuclein *via* altered interaction with lipid membranes in addition to lysosomal defects. It was proposed that GlcCer and another glycosphingolipid could induce toxic conversion of α-Synuclein (Suzuki et al., 2018). Recently, a study revealed that GBA deficiency may impact on the formation of α-Synuclein tetramers and related multimers (Kim et al., 2018). The major normal structure of α-Synuclein is a folded tetramer that is resistant to aggregation (Bartels et al., 2011). In comparison with tetramers, the monomer of α-Synuclein tends to aggregate and transition to insoluble deposits such as Lewy bodies (Bartels et al., 2011). Thus, the authors concluded that the accumulation of GlcCer due to GBA mutation destabilizes α-Synuclein tetramers and related multimers and increases the level of α-Synuclein monomers, eventually contributing to α-Synuclein aggregation and neurodegeneration in PD (Kim et al., 2018). Taken together, these studies highlight the mechanistic connection between GBA deficiency and α-Synuclein properties, providing unique therapeutic opportunities for reducing neurotoxicity of α-Synuclein for treatment of PD.

## Conclusions and Perspectives

In this review article, we summarized how phase transition, posttranslational modifications, physical and physiological status of α-Synuclein, inflammation, lipid metabolism, and genetic mutations may correlate with different functional consequences in PD (Figure 1). Proteostasis of α-Synuclein, as involved in maintaining normal cellular metabolic functions, plays a key role in the neurodegenerative process. The evolution and transformation of different species of α-Synuclein significantly affect the pathogenesis of PD.

Further work is required to elucidate the detailed mechanisms that regulate the proteostasis of α-Synuclein, particularly the initiating events of aggregation. Genetic mutations involved in early-onset familial PD may contribute to the phase transition of α-Synuclein, especially in the early stage of PD development; and mechanistic insight into this field may help develop novel therapeutic strategies to target α-Synucleinopathy. iPSC-derived dopaminergic neurons with LRRK2 G2019S mutation present with the accelerated accumulation of α-Synuclein. Treatment with terazosin, which can activate phosphoglycerate kinase 1 (PGK1) and subsequently increase cellular ATP level, reverses the elevation of α-Synuclein (Cai et al., 2019). Importantly, a lower frequency and slower progression of PD, and reduced disease-related complications are found in individuals taking the prescribed drug terazosin (Cai et al., 2019). The molecular mechanism remains elusive but one possibility is that ATP has property of a hydrotrope and can inhibit the formation and facilitate the dissolving of aggregates (Patel et al., 2017; Hayes et al., 2018). The study exemplifies that a molecule targeting the phase transition of α-Synuclein may slow down the progression of PD clinically. With the discovery of more molecules/drugs that target different aspects of α-Synuclein phase transition and with a better understanding of the genetic regulation of α-Synucleinopathy, new interventional opportunities are promised to emerge for treatment of PD.

## Author Contributions

DH, WZ, XW, and ZC wrote the manuscript together.

## Conflict of Interest

The authors declare that the research was conducted in the absence of any commercial or financial relationships that could be construed as a potential conflict of interest.
